# Prevalence of several somatic diseases depends on the presence and severity of obstructive sleep apnea

**DOI:** 10.1371/journal.pone.0192671

**Published:** 2018-02-23

**Authors:** Ragnhild L. Tveit, Sverre Lehmann, Bjørn Bjorvatn

**Affiliations:** 1 Department of Global Public Health and Primary Care, University of Bergen, Bergen, Norway; 2 Norwegian Competence Center for Sleep Disorders, Haukeland University Hospital, Bergen, Norway; 3 Section of Thoracic Medicine, Department of Clinical Science, University of Bergen, Bergen, Norway; Ospedale S. Corona, ITALY

## Abstract

**Study objectives:**

The objective was to investigate the prevalence of heart attack, angina pectoris, stroke, hypertension, diabetes mellitus, chronic obstructive pulmonary disease, asthma and obesity in relation to the presence and severity of obstructive sleep apnea.

**Methods:**

The sample consisted of 1887 patients, with mean age of 48.6 years (range 16–83 years), referred to a university hospital on suspicion of obstructive sleep apnea. The patients filled out a questionnaire asking whether they were previously diagnosed with the comorbidities in interest. Obstructive sleep apnea was diagnosed and categorized based on a standard respiratory polygraphic sleep study using a type 3 portable monitor. The patients’ weight, height and blood pressure were measured during the consultations.

**Results:**

In total, 37.9% were categorized as not having obstructive sleep apnea (Apnea-hypopnea index <5), 29.6% mild obstructive sleep apnea (Apnea-hypopnea index 5–14.9), 17.3% moderate obstructive sleep apnea (Apnea-hypopnea index 15–29.9), and 15.2% severe obstructive sleep apnea (Apnea-hypopnea index ≥30). The prevalence of heart attack, angina pectoris, hypertension, measured systolic blood pressure ≥140 mmHg, measured diastolic blood pressure ≥90 mmHg, diabetes mellitus and obesity (body mass index≥30) were higher with greater obstructive sleep apnea severity. Logistic and linear regression analyses showed that these comorbidities were positively associated with obstructive sleep apnea severity. This was not the case for stroke, chronic obstructive pulmonary disease and asthma. After adjustment for sex, age, alcohol and smoking in the logistic regression analyses, hypertension, measured systolic blood pressure ≥140 mmHg, measured diastolic blood pressure ≥90 mmHg and obesity remained positively associated with obstructive sleep apnea severity.

**Conclusions:**

A higher prevalence of heart attack, angina pectoris, hypertension, diabetes mellitus, and obesity was seen with greater obstructive sleep apnea severity. Obesity and hypertension, conditions easy to clinically assess, appear as the most central comorbidities with greater obstructive sleep apnea severity.

## Introduction

Obstructive sleep apnea (OSA) is a common, but often under-diagnosed disorder [1, 2]. The prevalence of OSA in the adult Norwegian population has been estimated to be 16% for apnea-hypopnea index (AHI) ≥5 and 8% for AHI≥15 [3]. OSA affects male individuals more commonly than female individuals [2, 4].

OSA has been recognized as an independent risk factor for cardiovascular disease. Morbidity such as stroke, myocardial infarction, unstable angina, hypertension and diabetes are associated with the disorder [5, 6]. Greater prevalence of OSA is seen in patients with coronary artery disease, compared to subjects without coronary artery disease [7]. It has also been reported that men with OSA have an increased risk of developing cardiovascular disease, independent of age, body mass index (BMI), blood pressure, and smoking [8]. The cardiovascular risk seen in patients with severe OSA is reduced with continuous positive airway pressure (CPAP) treatment according to Marin et al. [6].

Several studies show an association between OSA and hypertension [9–11], and OSA has been suggested as a possible risk factor for hypertension [10]. Haentjens et al.’s meta-analysis observed that CPAP treatment reduced blood pressure in patients with OSA, with a larger effect in severe OSA [12], indicating a correlation between hypertension and OSA. A dose-response relationship between OSA and blood pressure elevations independent of age, sex, BMI and other factors has been reported [10]. However, another study did not show a statistical difference once BMI was factored out. This implicated that the development of hypertension was more closely related to obesity than to OSA [13].

There is an increasing amount of evidence suggesting a link between OSA and insulin resistance and type 2 diabetes mellitus [7]. A link between OSA severity and the risk of type 2 diabetes mellitus, independently of obesity, is also suggested in epidemiological studies [14].

An increased incidence of stroke has been related to OSA. It has been reported that about half of patients who had an acute stroke have sleep-related disturbances [15]. The risk for developing stroke increases with increased severity of sleep apnea at baseline [7, 16]. Stroke and transient ischemic attack (TIA) are also risk factors for developing obstructive apnea [7].

Chronic obstructive pulmonary disease (COPD), asthma and COPD combined with asthma have been found to be more prevalent among OSA patients compared to matched controls [17]. A recently published Norwegian general population study suggests that women with asthma and chronic airflow limitation (assessed by spirometry with bronchodilator tests) are at particular risk for OSA [18]. Moreover, subjects suffering from the “overlap syndrome”, defined as co-existence of OSA and COPD, are at increased risk for exacerbations, pulmonary hypertension and death, which might be reduced by CPAP treatment [19]. However, The Sleep Heart Health Study reported that there was no association between mild obstructive airway disease and OSA [20].

According to World Health Organization’s (WHO) 10^th^ revision of the International Classification of Diseases (ICD-10) obesity is a diagnosis, and defined as BMI greater than or equal to 30 [21]. OSA is clearly associated with obesity [11, 14], and Franklin et al. list it as one of the key signs of OSA in female individuals [11]. The pathophysiology of OSA in obesity is considered to be multifactorial [14], where increased fat mass in obese individuals is a connection [22].

Patients with OSA are often subject to somatic comorbidity, such as those mentioned above. These conditions address different areas within medicine and there are few previous studies that consider the vast variety of comorbidities related to OSA within a reasonable sample size. Consequently, a study evaluating the association between several of the most common somatic comorbidities and OSA is warranted to enlighten the complexity of this patient group. With this in mind we investigated a large group of patients with suspected OSA referred to a major university hospital in Norway. Using a questionnaire the subjects’ somatic comorbidity was identified and clinically evaluated during the patient visit at the hospital. The main aim of this study was to investigate the prevalence of heart attack, angina pectoris, stroke, hypertension, diabetes mellitus, COPD, asthma and obesity in relation to the presence and severity of OSA.

## Materials and methods

The total sample consisted of 1929 patients referred to Haukeland University Hospital on suspicion of obstructive sleep apnea during the period of 2011 to 2014. Due to no objective registrations, 42 individuals were excluded, thus the final sample included 1887 patients. The mean age was 48.6 years (range 16–83 years) and 70.6% were men.

As described in detail previously [23], the patients underwent a standard respiratory sleep study to diagnose whether they had OSA. This was done using a type 3 portable monitor. The scoring rules were in accordance with the 2007 American Academy of Sleep Medicine manual. Apneas were defined as a reduction of 90% or more of baseline nasal airflow with a duration of at least 10 seconds. Hypopneas were defined as a nasal flow reduction of 30–90% of baseline, lasting at least 10 seconds accompanied by an oxygen desaturation of ≥ 4%. OSA was diagnosed and classified according to AHI as no OSA (<5), mild (5–14.9), moderate (15–29.9) or severe (≥30) OSA. The vast majority of the sleep recordings took place in the patients’ home. The rest of the patients slept in a hospital hotel. The patient-reported time period before sleep onset and after morning awakening were excluded from the sleep record before manual scoring [23].

The patients filled out a questionnaire prior to the respiratory sleep study, S1 and S2 Survey questions. A nurse and/or doctor revised the questionnaire during the consultation to make sure the answers delivered were reliable. Changes were made if necessary.

The patients were asked about their current smoking habits (number of cigarettes per day). For data analyses purposes their answers were categorized into a “no smoking” and “smoking” variable. The latter included 1 and more cigarettes per day.

The patients’ alcohol consumption was classified as daily, 3–5 days per week, 1–2 days per week, rarely or never. An “alcohol” variable was generated for data analyses, combining daily consumption and 3–5 days per week. The “no alcohol” variable included 1–2 days per week, rarely and never. This dichotomy was created to ease the analyses and differentiate between alcohol consumption on a regular basis or rarely.

The patients were asked about whether they were previously diagnosed with heart attack (yes/no), stroke (yes/no), diabetes mellitus (yes/no), hypertension (yes/no), angina pectoris (yes/no), COPD (yes/no) and asthma (yes/no). Furthermore, the patients were asked whether they currently received treatment with medication for diabetes mellitus, hypertension, angina pectoris, COPD and asthma.

During the consultation the patients’ sex and age were noted, and weight (kg) and height (m) measured. Their BMI was calculated by weight in kilograms divided by squared height in meters. Obesity was defined as BMI greater than or equal to 30.

The patients’ blood pressure was measured by a nurse and/or doctor during the consultation. Prior to the measurement the patient sat still for 10 minutes. The blood pressure was then measured using an automatic, cuff-style, bicep monitor. Two recordings were done and the second recording was selected. In case of strongly deviating results, the blood pressure was measured manually.

The study was approved by the Ethics Board (The Regional Committee for Medical and Health Related Ethics in Western Norway). Written informed consent was obtained from all individual participants included in the study.

### Statistics

The data analyses were performed using IBM SPSS Statistics version 22. Pearson chi-square tests were used to evaluate the differences in the patients’ characteristics according to the presence and severity of OSA. Thereafter, bivariate logistic regression (crude) analyses were performed with OSA severity (0 = AHI <15, 1 = AHI ≥15) as the dependent variable. Separate multivariate logistic regression analyses (adjusted) were then done for each characteristic together with age and sex as co-variates. The adjusted analyses were repeated, including smoking and alcohol in addition to age and sex as co-variates. Linear regressions with AHI as the dependent variable were also performed. The comorbidity parameters of interest were further used in crude logistic regression analyses as dependent variables, using OSA severity (mild, moderate, severe) as co-variate. The significance level was set to 0.05.

## Results

Among the patients referred on suspicion of OSA 37.9% had a normal AHI (<5), yielding a prevalence rate of OSA of 62.1%. Out of the referred patients 29.6% had mild OSA, 17.3% had moderate OSA, and 15.2% had severe OSA (Table 1).

**Table 1:** Characteristics of patients (n = 1887) referred to a university hospital with suspicion of obstructive sleep apnea.

	No OSA	Mild OSA	Moderate OSA	Severe OSA	Chi-square p-value
Patients, % (n)	37.9 (716)	29.6 (558)	17.3 (327)	15.2 (286)	
Sex, males, % (n)	63.3 (453)	67.7 (378)	79.5 (260)	84.3 (241)	<.*0005*
Smoking, % (n)	27.8 (187)	24.5 (129)	21.3 (66)	19.7 (52)	*0*.*03*
Alcohol ≥3d/week % (n)	5.9 (41)	6.4 (35)	10.0 (32)	10.0 (28)	*0*.*03*
Heart attack, % (n)	2.6 (18)	5.0 (27)	7.3 (23)	9.0 (25)	<.*0005*
Angina pectoris, % (n)	2.8 (19)	3.2 (17)	6.3 (19)	6.6 (18)	.*009*
Stroke, % (n)	2.3 (16)	2.3 (12)	3.6 (11)	3.4 (9)	.56
Hypertension, % (n)	22.8 (156)	35.2 (189)	41.3 (131)	53.3 (147)	<.*0005*
SBP ≥ 140 mmHg, % (n)	22.7 (139)	33.7 (169)	38.7 (113)	41.4 (110)	<.*0005*
DBP≥ 90 mmHg, % (n)	14.2 (87)	24.0 (120)	29.1 (85)	35.1 (93)	<.*0005*
Diabetes mellitus, % (n)	7.4 (51)	7.1 (38)	12.2 (39)	13.8 (38)	.*001*
COPD, % (n)	3.2 (22)	3.6 (19)	4.8 (15)	3.3 (9)	.66
Asthma, % (n)	19.2 (131)	17.4 (93)	13.4 (42)	16.1 (44)	.15
Obesity, BMI ≥30, % (n)	30.1 (197)	44.6 (232)	41.0 (126)	64.7 (180)	<.*0005*

SBP: Measured systolic blood pressure, DBP: Measured diastolic blood pressure,

COPD: chronic obstructive pulmonary disease, BMI: body mass index.

Being male and alcohol consumption were associated with greater OSA severity. Smoking was associated with lower OSA severity (Table 1).

The prevalence of heart attack, angina pectoris, hypertension, diabetes, and obesity were significantly higher with greater OSA severity (Table 1). This was not the case for stroke, COPD, or asthma. Measured systolic blood pressure ≥140 mmHg and diastolic blood pressure ≥90 mmHg also showed a higher prevalence with greater OSA severity (Table 1).

When only including the patients who received treatment with medication for the particular comorbidity, diabetes and hypertension showed a significant increase in prevalence with greater OSA severity (Table 2). Angina pectoris with treatment showed a similar increase, but this was not of significance. COPD and asthma with treatment were not associated with OSA (Table 2).

**Table 2:** Angina pectoris, hypertension, diabetes mellitus, COPD and asthma treated with medication among patients (n = 1887) referred to a university hospital with suspicion of obstructive sleep apnea.

	No OSA	Mild OSA	Moderate OSA	Severe OSA	Chi-square p-value
Angina pectoris, treated with medication, % (n)	1.8 (12)	2.8 (15)	4.0 (12)	4.8 (13)	.06
Hypertension, treated with medication, % (n)	16.7 (112)	24.0 (126)	33.3 (105)	44.5 (121)	<.*0005*
Diabetes mellitus, treated with medication, % (n)	5.2 (36)	4.7 (25)	9.7 (31)	10.9 (30)	<.*0005*
COPD, treated with medication, % (n)	2.3 (16)	2.6 (14)	3.8 (12)	2.9 (8)	.61
Asthma, treated with medication, % (n)	11.4 (76)	11.0 (57)	9.6 (30)	10.0 (27)	.82

COPD: chronic obstructive pulmonary disease.

Table 3 presents the results from the logistic regression analyses with OSA severity (AHI ≥15) as the dependent variable. Male sex, age, alcohol consumption, heart attack, angina pectoris, hypertension, systolic blood pressure ≥140 mmHg, diastolic blood pressure ≥90 mmHg, diabetes mellitus and obesity were all positively associated with OSA severity. Smoking was however negatively associated with OSA severity in the crude analyses. Asthma showed a non-significant negative association with OSA severity. In the multivariate (adjusted) regression analyses hypertension, systolic blood pressure ≥140 mmHg, diastolic blood pressure ≥90 mmHg, diabetes mellitus and obesity remained positively associated when adjusted for age and sex. Similarly, when adjusted for age, sex, alcohol and smoking, hypertension, systolic blood pressure ≥140 mmHg, diastolic blood pressure ≥90 mmHg and obesity remained positively associated with OSA severity. Furthermore, when BMI was included as co-variate together with age, sex, alcohol and smoking, diastolic blood pressure remained positively associated with OSA severity (OR = 1.55, CI = 1.19–2.02), and in addition, COPD became negatively associated with OSA severity (OR = 0.54, CI = 0.30–0.97).

**Table 3:** Logistic regression analyses with moderate to severe obstructive sleep apnea (OSA with AHI ≥15) as the dependent variable among patients (n = 1887) referred to a university hospital with suspicion of obstructive sleep apnea.

	Moderate-severe OSA		
Characteristics (n in crude analysis)	Crude OR (95% CI)	Adjusted OR (95% CI)^a^ n = 1669–1839	Adjusted OR (95% CI)^b^ n = 1559–1723
Sex			
Female (555)	1.00		
Male (1332)	*2*.*39 (1*.*89–3*.*02)*		
Age (1887)	*1*.*05 (1*.*04–1*.*06)*		
Alcohol			
No (1703)	1.00	1.00	
Yes (136)	*1*.*70 (1*.*19–2*.*42)*	1.19 (0.82–1.73)	
Smoking			
No (1339)	1.00	1.00	
Yes (434)	*0*.*72 (0*.*57–0*.*92)*	0.89 (0.69–1.15)	
Heart attack			
No (1723)	1.00	1.00	1.00
Yes (93)	*2*.*30 (1*.*51–3*.*50)*	1.08 (0.69–1.69)	1.02 (0.64–1.63)
Angina pectoris			
No (1696)	1.00	1.00	1.00
Yes (73)	*2*.*21 (1*.*38–3*.*53)*	1.33 (0.81–2.19)	1.34 (0.79–2.27)
Stroke			
No (1741)	1.00	1.00	1.00
Yes (48)	1.53 (0.85–2.73)	0.83 (0.45–1.54)	0.82 (0.44–1.54)
Hypertension			
No (1192)	1.00	1.00	1.00
Yes (623)	*2*.*24 (1*.*83–2*.*75)*	*1*.*54 (1*.*23–1*.*93)*	*1*.*57 (1*.*24–1*.*98)*
SBP ≥ 140			
No (1139)	1.00	1.00	1.00
Yes (531)	*1*.*74 (1*.*40–2*.*15)*	*1*.*30 (1*.*03–1*.*64)*	*1*.*28 (1*.*01–1*.*63)*
DBP ≥ 90			
No (1284)	1.00	1.00	1.00
Yes (385)	*2*.*05 (1*.*63–2*.*59)*	*1*.*83 (1*.*43–2*.*35)*	*1*.*81 (1*.*40–2*.*34)*
Diabetes mellitus			
No (1654)	1.00	1.00	1.00
Yes (166)	*1*.*90 (1*.*38–2*.*63)*	*1*.*45 (1*.*02–2*.*06)*	1.39 (0.97–1.98)
COPD			
No (1736)	1.00	1.00	1.00
Yes (65)	1.22 (0.73–2.04)	0.68 (0.39–1.18)	0.67 (0.38–1.18)
Asthma			
No (1492)	1.00	1.00	1.00
Yes (310)	0.76 (0.58–1.00)	0.87 (0.65–1.16)	0.91 (0.68–1.22)
Obesity, BMI ≥30			
No (1024)	1.00	1.00	1.00
Yes (735)	*1*.*91 (1*.*56–2*.*33)*	*2*.*24 (1*.*80–2*.*79)*	*2*.*29 (1*.*83–2*.*87)*

^a^: Adjusted for age and sex,

^b^: Adjusted for age, sex, smoking and alcohol, BMI: body mass index, SBP: Measured systolic blood pressure, DBP: Measured diastolic blood pressure, COPD: chronic obstructive pulmonary disease.

Linear regressions with AHI as dependent variable showed a positive association with heart attack (B = 7.45, std.error = 1.87, beta = 0.093, t = 3.98, p<0.0005), angina pectoris (B = 5.71, std.error = 2.11, beta = 0.064, t = 2.71, p = 0.007), hypertension (B = 7.52, std.error = 0.86, beta = 0.20, t = 8.80, p<0.0005), measured systolic blood pressure ≥140 mmHg (B = 5.87, std.error = 0.92, beta = 0.16, t = 6.41, p<0.0005), measured diastolic blood pressure ≥90 mmHg (B = 7.39, std.error = 1.01, beta = 0.18, t = 7.31, p<0.0005), diabetes mellitus (B = 5.23, std.error = 1.43, beta = 0.085, t = 3.65, p<0.0005), and obesity (B = 8.52, std.error = 0.83, beta = 0.24, t = 10.27, p<0.0005). Linear regressions with AHI as dependent variable showed no association with stroke (B = 1.00, std.error = 2.58, beta = 0.009, t = 0.39, p = 0.70), COPD (B = -0.037, std.error = 2.23, beta = 0.00, t = -0.017, p = 0.99) and asthma (B = -1.95, std.error = 1.10, beta = -0.042, t = -1.77, p = 0.077).

The results from logistic regression analyses using the comorbidity parameters as dependent variables and OSA severity (mild, moderate, severe) as co-variate are shown in Table 4. Increasing OSA severity was associated with higher prevalence of heart attack, angina pectoris, hypertension, diabetes mellitus and obesity with mild OSA being the reference.

**Table 4:** Logistic regression analyses with heart attack, angina pectoris, stroke, hypertension, diabetes mellitus, COPD, asthma, and obesity as the dependent variables and OSA severity as co-variate among patients (n = 1887) referred to a university hospital with suspicion of obstructive sleep apnea.

	Heart attack	Angina pectoris	Stroke	Hypertension	Diabetes mellitus	COPD	Asthma	Obesity, BMI ≥30
	Crude OR (95% CI)	Crude OR (95% CI)	Crude OR (95% CI)	Crude OR (95% CI)	Crude OR (95% CI)	Crude OR (95% CI)	Crude OR (95% CI)	Crude OR (95% CI)
OSA								
Mild (n = 520–537)	1.00	1.00	1.00	1.00	1.00	1.00	1.00	1.00
Moderate (n = 305–319)	1.48	*1*.*99*	1.60	1.30	*1*.*82*	1.36	0.73	0.86
	(0.83–2.63)	*(1*.*02–3*.*89)*	(0.70–3.67)	(0.98–1.73)	*(1*.*14–2*.*92)*	(0.68–2.71)	(0.49–1.09)	(0.65–1.15)
Severe (n = 268–278)	*1*.*86*	*2*.*12*	1.50	*2*.*10*	*2*.*10*	0.92	0.91	*2*.*28*
	*(1*.*06–3*.*28)*	*(1*.*07–4*.*18)*	(0.62–3.60)	*(1*.*56–2*.*82)*	*(1*.*30–3*.*37)*	(0.41–2.07)	(0.61–1.35)	*(1*.*69–3*.*08)*

COPD: chronic obstructive pulmonary disease, BMI: body mass index.

## Discussion

Among patients referred on suspicion of OSA there was a higher prevalence of heart attack, angina pectoris, hypertension, diabetes mellitus, and obesity with greater OSA severity. This was also the case for measured systolic blood pressure ≥140 mmHg and diastolic blood pressure ≥90 mmHg. However, there was no significant association between OSA severity and stroke, COPD and asthma.

Hypertension, measured systolic blood pressure ≥140 mmHg and measured diastolic blood pressure ≥90 mmHg proved to be significantly associated with OSA severity also after adjusting for age, sex, smoking and alcohol. These findings support other studies [9–11], though not all include adjustment for smoking and alcohol [13]. Hypertension, measured systolic blood pressure ≥140 mmHg and measured diastolic blood pressure ≥90 mmHg were also statistically significant unique contributors to AHI in the linear regression analyses. This suggests that hypertension is clearly associated with OSA severity.

Obesity has an indisputable association with OSA [11, 14], also seen in our results. The relationship between AHI and hypertension has also been suggested to be a result of obesity [13]. However, in our study diastolic blood pressure remained significantly associated with OSA severity also when BMI was included as co-variate.

As seen in Table 4 and the linear regressions there was a significant association between OSA severity and heart attack and angina pectoris. However, the two conditions were not associated with OSA severity after adjustment with age and sex. This is in contrast to results from the Sleep Heart Health Study where a significant association of AHI with incident coronary heart disease was seen in men, however, not in women when adjusting for age [24]. Interestingly, a recent study found no relationship between OSA severity and diagnosed cardiometabolic disease [25].

Fredheim et al. reported that extremely obese patients with type 2 diabetes and prediabetes have higher odds of OSA, even after adjustment for amongst others age, sex, smoking and alcohol consumption [26]. This is somehow in contrast to our findings showing that diabetes mellitus did not remain significantly associated with OSA after adjusting for age, sex, smoking and alcohol. However, Fredheim et al. used glucose tolerance tests to identify cases, and also in our study we found a clear association between diabetes and AHI in the linear regression analyses with increasing prevalence with greater OSA severity.

COPD and asthma were not associated with OSA severity in our study. This is in contrast to Greenberg-Dotan et al.’s findings [17]. Steveling et al. have previously reported that BMI and smoking (pack years) were the only predictors for OSA in a group of COPD patients [27]. COPD patients often experience a reduction in BMI with increasing severity of COPD (GOLD stages). Our study also reports that obesity (BMI ≥30) has a significant association with OSA severity. When BMI was included as a co-variate in our logistic regression analyses COPD was negatively associated with OSA severity. It could be of interest in future studies to examine the prevalence of OSA in the different GOLD stages, preferably with a study design avoiding as much selection bias as possible. Subjects with obstructive lung diseases frequently experience sleep disordered breathing [28], which might be perceived as asthma or COPD symptoms only, thus keeping them away from adequate OSA diagnostics.

In our study stroke was not associated with OSA severity. This is in contrast to Redline et al.’s findings that increasing OSA severity was significantly associated with increased stroke risk, even after adjustment for amongst others BMI, smoking, systolic blood pressure and diabetes [16]. Their study was a longitudinal cohort study, using polysomnography instead of a type 3 portable monitor.

Diabetes and hypertension showed a significant increase in prevalence with greater OSA severity among the patients receiving medication for the particular comorbidity. These results correlated with the results from the total patient group. This is of interest as treatment for the particular comorbidity can indicate severity of the comorbidity. On the other hand, medication will benefit the patient and a well-medicated disease may provide a healthier individual than without medication. Regarding this, OSA has been reported particularly prevalent in drug-resistant hypertension [29]. Our results with patients receiving medications should however be interpreted with caution, as it is difficult to draw conclusions as to what they indicate.

As seen in other studies, being male was positively associated with OSA severity [2, 4]. Surprisingly, there was a negative association between smoking and OSA severity. A possible explanation could be that smoking has become a well-known health hazard, possibly giving the patient increased incentive to quit smoking and easier for doctors to address to their patients. Smoking is considered a possible, but not established risk factor for OSA [2]. In contrast to our results Wetter et al. discovered a dose-response relationship between smoking and severity of sleep apnea [30].

A variety of associations between OSA and alcohol consumption have been reported [2, 31], and in our study alcohol was positively associated with OSA severity. Whereas smoking has become easier to address to patients over the years, alcohol consumption is perhaps less easy to address.

In our study, we chose to treat obesity as a disorder. Due to this BMI was not included as a risk factor, nor adjusted for in the analyses. Several of the comorbidities in interest are included in the metabolic syndrome, a cluster of risk factors for cardiovascular disease. While having slightly different definitions [32], metabolic syndrome covers amongst others abdominal obesity, hypertension and hyperglycemia [33]. OSA can be perceived in association with metabolic syndrome. Adjusting for only one of the components of the metabolic syndrome would be artificial, while adjusting for all components would most likely leave no significant associations.

There are various strengths and limitations to the present study. An important advantage was the study’s large sample size, giving weight to the results. Another strength was that a nurse and a doctor revised the questionnaire filled out by the patient during the consultation, and made changes if necessary. This functioned as a quality control of the answers filled out, in the case the patient had misunderstood the question. Measuring blood pressure and calculating BMI from height and weight provided objective information, hence strengthening the study. Another asset was that we studied the association between OSA severity and several somatic comorbidities treated with medication. Furthermore, both logistic and linear regressions were conducted, adding weight to the findings. One of the limitations to our study was that the somatic diagnoses were based on self-report. This gives room for human error such as the patient delivering misunderstood, subjective or false answers, not detected by the nurse or doctor revising the answers. Another limitation was that the diagnoses proclaimed by the patient were not controlled or set by the means of blood samples, lung function tests or a complete clinical examination. It can also be mentioned that our study did not differentiate between diabetes mellitus type 1 and type 2. The difference in pathogenesis between the two types of diabetes mellitus could indicate different associations with OSA severity. The dichotomy for alcohol consumption also serves as a limitation. Alcohol consumption has a cultural aspect, complicating the classification of “normal” and “excessive” alcohol consumption. This study was based on patients referred to a University hospital on suspicion of OSA. Hence, selection bias may have been introduced compared to the distribution of co-morbid diseases to OSA in the community. However, close to all patients in this region of Norway need to be referred to this hospital for CPAP treatment. It is therefore likely that the results are generalizable to other patients referred to hospital settings with suspicion of OSA, as mentioned previously by Bjorvatn et al. [23]. Our study design may have resulted in an under-selection of subjects suffering from conditions with overlapping symptoms with OSA, such as asthma and COPD. In addition, it is important to note that the “No OSA”-group was not equivalent to a healthy, general population. As mentioned previously in another study [23], a limitation was related to using polygraphy, and not polysomnography, for diagnosing OSA. AHI is underestimated in polygraphic registrations compared to polysomnography [34, 35]. However, we assume that the association between OSA severity and these somatic disorders would be similar with polysomnography, but such studies should be performed.

In conclusion, our results emphasize that there is a higher prevalence of heart attack, angina pectoris, hypertension, diabetes mellitus, and obesity with greater OSA severity. However, there was no association between OSA severity and stroke, COPD and asthma in our patient group. Obesity and hypertension, as well as measured systolic blood pressure ≥140 mmHg and diastolic blood pressure ≥90 mmHg, seemed to be central comorbidities with greater OSA severity. These conditions are easy to assess in every level of the health system and should be considered when faced with patients presenting symptoms of OSA.

## Supporting information

S1 Survey questions Norwegian version of survey questions used in study. (DOC)

S2 Survey questions English version of survey questions used in study. (DOC)
