# Supplementary material for: Prevalence of several somatic diseases depends on the presence and severity of obstructive sleep apnea
Source: PLoS One. 2018 Feb 23;13(2):e0192671. doi: 10.1371/journal.pone.0192671 (PMC5825017; doi:10.1371/journal.pone.0192671)
Supplement: S1 Survey questions — (DOC) [file pone.0192671.s001.doc]

**S1 Survey questions. Norwegian version of survey questions used in the study.**

Hvor mye røyker du (antall sigaretter per dag)? _____

Angi ditt alkoholforbruk:

daglig ❒ 3-5 dager/uke ❒ 1-2 dager/uke ❒ sjeldent ❒ aldri ❒

Har du tidligere fått påvist?

Hjerteinfarkt Ja ❒ Nei ❒

Hjerneslag Ja ❒ Nei ❒

Har du tidligere fått påvist? Får du *medikamentell* behandling

for dette nå?

Diabetes mellitus/sukkersyke Ja ❒ Nei ❒ Ja ❒ Nei ❒

Hypertensjon/ høyt blodtrykk Ja ❒ Nei ❒ Ja ❒ Nei ❒

KOLS Ja ❒ Nei ❒ Ja ❒ Nei ❒

Astma Ja ❒ Nei ❒ Ja ❒ Nei ❒

Angina pectoris Ja ❒ Nei ❒ Ja ❒ Nei ❒
