# Supplementary material for: Prevalence of several somatic diseases depends on the presence and severity of obstructive sleep apnea
Source: PLoS One. 2018 Feb 23;13(2):e0192671. doi: 10.1371/journal.pone.0192671 (PMC5825017; doi:10.1371/journal.pone.0192671)
Supplement: S2 Survey questions — (DOC) [file pone.0192671.s002.doc]

**S2 Survey questions. English version of survey questions used in the study.**

How many cigarettes do you smoke per day? _____

Describe your alcohol consumption:

daily ❒ 3-5 days/week ❒ 1-2 days/week ❒ rarely ❒ never ❒

Have you previously been diagnosed with?

Heart attack Yes ❒ No ❒

Stroke Yes ❒ No ❒

Have you previously been diagnosed with? Are you treated with *medication* for this now?

Diabetes mellitus Yes ❒ No ❒ Yes ❒ No ❒

Hypertension Yes ❒ No ❒ Yes ❒ No ❒

COPD Yes ❒ No ❒ Yes ❒ No ❒

Asthma Yes ❒ No ❒ Yes ❒ No ❒

Angina pectoris Yes ❒ No ❒ Yes ❒ No ❒
